# Gibberellin Promotes Shoot Branching in the Perennial Woody Plant *Jatropha curcas*

**DOI:** 10.1093/pcp/pcv089

**Published:** 2015-06-15

**Authors:** Jun Ni, Congcong Gao, Mao-Sheng Chen, Bang-Zhen Pan, Kaiqin Ye, Zeng-Fu Xu

**Affiliations:** ^1^School of Life Sciences, University of Science and Technology of China, Hefei, Anhui 230027, China; ^2^Key Laboratory of Tropical Plant Resources and Sustainable Use, Xishuangbanna Tropical Botanical Garden, Chinese Academy of Sciences, Menglun, Yunnan 666303, China

**Keywords:** Axillary bud, Bud outgrowth, Cytokinin, Gibberellin, Shoot branching, Strigolactone

## Abstract

Strigolactone (SL), auxin and cytokinin (CK) interact to regulate shoot branching. CK has long been considered to be the only key phytohormone to promote lateral bud outgrowth. Here we report that gibberellin also acts as a positive regulator in the control of shoot branching in the woody plant *Jatropha curcas*. We show that gibberellin and CK synergistically promote lateral bud outgrowth, and that both hormones influence the expression of putative branching regulators, *J. curcas BRANCHED1* and *BRANCHED2*, which are key transcription factors maintaining bud dormancy. Moreover, treatment with paclobutrazol, an inhibitor of de novo gibberellin biosynthesis, significantly reduced the promotion of bud outgrowth by CK, suggesting that gibberellin is required for CK-mediated axillary bud outgrowth. In addition, SL, a plant hormone involved in the repression of shoot branching, acted antagonistically to both gibberellin and CK in the control of lateral bud outgrowth. Consistent with this, the expression of *JcMAX2,* a *J. curcas* homolog of *Arabidopsis MORE AXILLARY GROWTH 2* encoding an F-box protein in the SL signaling pathway, was repressed by gibberellin and CK treatment. We also provide physiological evidence that gibberellin also induces shoot branching in many other trees, such as papaya, indicating that a more complicated regulatory network occurs in the control of shoot branching in some perennial woody plants.

## Introduction

Plant shoot architecture is determined by the primary apical meristem and the derived lateral branches. In many crops, the number of lateral branches has a profound effect on yield (Kebrom et al. 2013). For decades, intensive research has focused on identifying and characterizing the phytohormones that control lateral bud outgrowth. How plants flexibly optimize their architecture to adapt to the changing environment through manipulation of their endogenous phytohormone balance remains largely unknown.

Depending on environmental conditions, axillary buds either develop into lateral branches or remain dormant. Earlier studies showed that basipetally transported auxin inhibits lateral bud outgrowth (Thimann and Skoog 1934, Morris 1977), and that interactions between auxin and cytokinin (CK) play a central role in the establishment of apical dominance (Wickson and Thimann 1958, Scott et al. 1967). However, auxin does not directly inhibit bud outgrowth, since apically supplied auxin does not travel into the lateral bud after decapitation (Hall and Hillman 1975). This result supports the hypothesis that auxin controls lateral bud outgrowth in conjunction with secondary messengers (Sachs and Thimann 1967, Bangerth 1994, Li et al. 1995). CK was postulated to function as a second messenger that relays the auxin signal into the lateral buds (Snow 1937, Bangerth 1994, Leyser 2003). Auxin can directly inhibit CK biosynthesis through the *AXR1*-dependent auxin signaling pathway (Nordström et al. 2004), and CK is the only identified phytohormone to date known to control lateral bud outgrowth positively in pea (*Pisum sativum*) and *Arabidopsis thaliana*. Indeed, a few hours after decapitation, the CK level increased several fold in the lateral buds of pea and chickpea (Turnbull et al. 1997, Tanaka et al. 2006). Tanaka et al. (2006) found that the expression level of the pea gene *adenosine phosphate isopentenyltransferase* (*PsIPT*), encoding a key enzyme in CK biosynthesis, was increased at the node after decapitation, and proposed that lateral bud outgrowth after decapitation was due to locally increased accumulation of CK. Recently, an excellent study by Mason et al. (2014) showed that sucrose acts as the second messenger that transduces the decapitation signal, and highlighted the specific role of sugar in the control of lateral bud outgrowth.

It has long been postulated that a carotenoid-derived root signal inhibits shoot branching and acts as a graft-transmissible factor that moves up the shoot and is required for auxin-mediated repression of lateral bud outgrowth (Beveridge et al. 1994, Beveridge et al. 1997, Morris et al. 2001). This signal was identified as strigolactones (SLs), a group of phytohormones discovered relatively recently (Gomez-Roldan et al. 2008, Umehara et al. 2008). SLs act downstream of auxin to inhibit lateral bud outgrowth (Brewer et al. 2009, Agusti et al. 2011). Direct application of GR24 (an analog of SL) efficiently suppresses the lateral bud outgrowth induced by decapitation or CK treatment (Brewer et al. 2009, Dun et al. 2012).

Gibberellins are a group of key hormones regulating many aspects of plant growth and development (Olszewski et al. 2002, Yamaguchi 2008). In pea, gibberellin seems to play an inhibitory role in lateral bud outgrowth (Scott et al. 1967). In Arabidopsis, a gibberellin-insensitive (*gai*) mutant shows a reduction in apical dominance and an increased number of axillary shoots (Koornneef et al. 1985). In turfgrass and *Populus* trees, overexpression of the gibberellin-catabolizing gene *GA2ox* led to an increased number of tillers or branches, suggesting that gibberellin may also play a negative role in the control of shoot branching in these species (Agharkar et al. 2007, Mauriat et al. 2011, Zawaski and Busov 2014). However, stimulation by gibberellin of axillary bud development was reported in citrus and snapdragon (Marth et al. 1956) and in sweet cherry (Elfving et al. 2011). The involvement of gibberellin biosynthesis in the light effect on bud burst was also demonstrated in rose (Choubane et al. 2012). Here we present evidence that gibberellin is a positive regulator in controlling shoot branching in the perennial woody plant *Jatropha curcas*, a promising biofuel feedstock (Sato et al. 2011, Chen et al. 2014, Wu et al. 2015). We also investigated the interactions between gibberellin and CK in the control of lateral bud outgrowth.

## Results

### Gibberellin treatment promotes lateral bud outgrowth

**Fig. 1:**
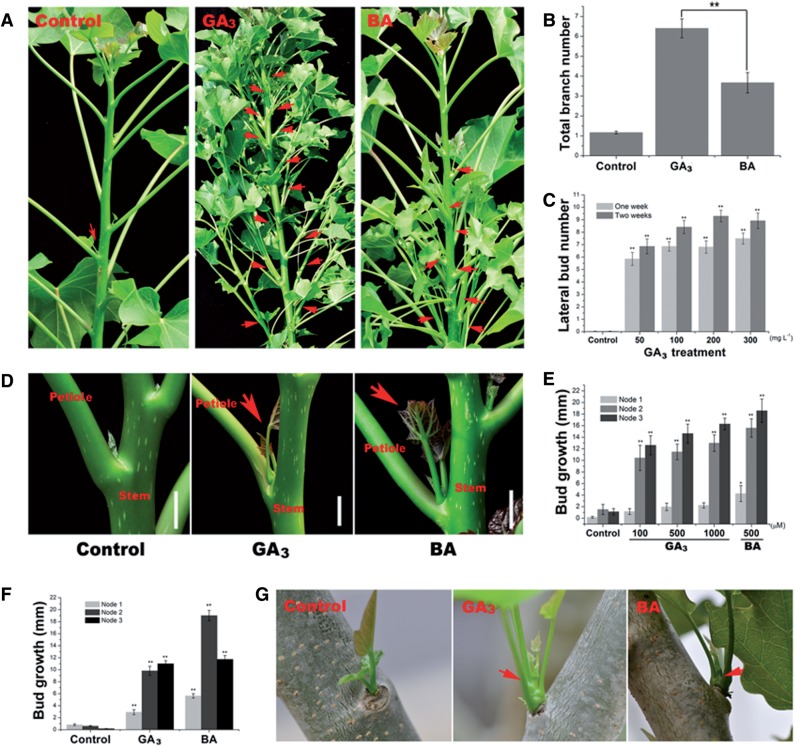
GA_3_ and BA promote shoot branching in *J. curcas*. (A) Shoot branching stimulated by GA_3_ or BA treatment in the young stems of 2-year-old *J. curcas* trees. (B) The number of induced branches was counted 4 weeks after GA_3_ (500 mg l^–1^) or BA (500 mg l^–1^) treatment (*n* = 30). (C) The number of stimulated axillary buds was counted 1 and 2 weeks after the application of various concentrations of GA_3_ to the top region of the branch (∼20 cm; *n* = 28–45). (D) Representative lateral bud stimulated by GA_3_ treatment in 2-year-old *J. curcas* trees. (E) Axillary bud length at nodes 1–3 of 4-week-old *J. curcas* seedlings measured 2 weeks after GA_3_ or BA treatment (*n* = 12–14). (F) Buds at nodes 1–3 of 5-week-old *J. curcas* seedlings showed different responsiveness to GA_3_ or BA (500 μM) treatment (*n* > 20). (G) Lateral bud on an old stem of a 3-year-old *J. curcas* tree, stimulated by GA_3_ or BA (500 μM) treatment. Values are means ± SE for (B), (C), (E) and (F). Student’s *t*-test was used to determine significant differences between the indicated groups in (B) or between the treated and control groups in (C), (E) and (F). Significance levels: *P < 0.05; **P < 0.01. Red arrows indicate the stimulated axillary buds.

We found that lateral branch outgrowth could be efficiently stimulated by treatment with GA_3_ or the synthetic CK, 6-benzyladenine (BA), in 2-year-old *J. curcas* trees (Fig. 1A). Interestingly, GA_3_ was more effective at promoting shoot branching (Fig. 1A, B). We then investigated how the axillary buds respond to different concentrations of GA_3_. Treatment with higher concentrations of GA_3_ led to an increased number of stimulated lateral buds (Fig. 1C), although it caused severe side effects, such as shoot apex necrosis. Typically, lateral branches induced by GA_3_ or BA treatment had an actively growing stem with several small leaves (Fig. 1D).

GA_3_ and BA were also applied to the shoots of 4-week-old *J. curcas* seedlings. Approximately 2–3 d after treatment, the axillary buds had undergone marked outgrowth (Fig. 1E), and treatment with BA promoted bud outgrowth to a greater extent than did GA_3_ (Fig. 1E).

In pea, axillary buds at different locations vary in responsiveness to CK treatment and decapitation (King and Vanstaden 1988, Morris et al. 2005). We investigated the responsiveness to GA_3_ and BA treatment of axillary buds located at different positions in 5-week-old *J. curcas* seedlings. Whereas all buds were successfully stimulated by the treatments, those located at higher positions (nodes 2 and 3) were the most responsive (Fig. 1F).

Axillary buds located on old stems are dormant and seldom develop into branches in *J. curcas* (Ghosh et al. 2011). We investigated whether axillary buds on old stems (approximately 1.5 m above the ground) of 3-year-old *J. curcas* trees were also sensitive to GA_3_ or BA treatment. We detected marked outgrowth of the axillary buds 1 week after GA_3_ or BA treatment (Fig. 1G), suggesting that the dormant axillary buds located on old stems can still be activated by these phytohormones.

We further generated transgenic *J. curcas* overexpressing a gibberellin biosynthesis gene, *JcGA20ox1* (GenBank accession No. KM454465), which encodes a gibberellin 20-oxidase in *J. curcas* (Supplementary Fig. S1). Since the transgenic shoots failed to produce roots, which probably resulted from the increased gibberellin level produced by overexpression of the transgene *JcGA20ox1*, they were grafted onto rootstocks of wild-type *J. curcas* seedlings. The grafted *JcGA20ox1* transgenic plants showed accelerated growth and stem elongation (Supplementary Fig. S1C), and enhanced lateral bud outgrowth (Supplementary Fig. S1D). An enhanced branching phenotype was observed in the young transgenic trees a few months after transplantation in the field (Supplementary Fig. S1E). These results support our finding that gibberellin promotes lateral bud outgrowth.

### Interactions of gibberellin and CK in the control of lateral bud outgrowth

**Fig. 2:**
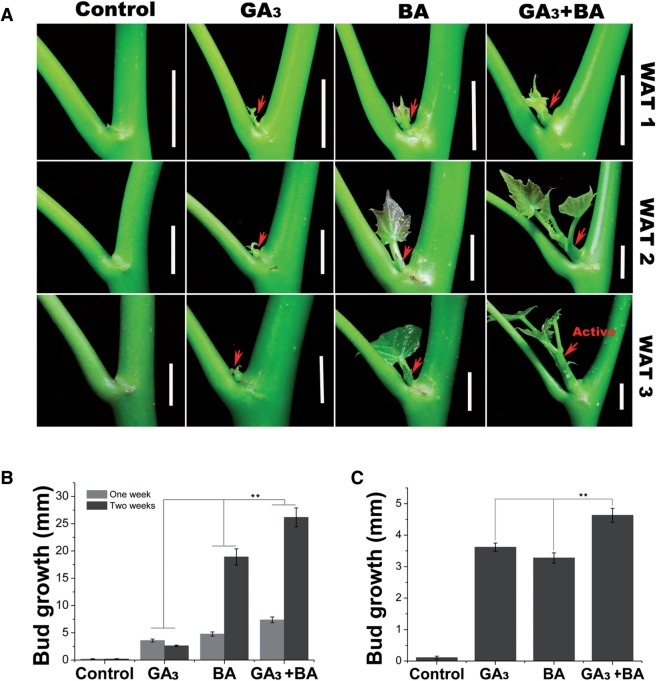
GA_3_ and BA synergistically stimulate bud outgrowth in 3-week-old seedlings of *J. curcas*. (A) Photographs showing representative outgrowth of the buds at node 1 at 1, 2 and 3 weeks after treatment (WAT 1, 2 and 3), respectively. Red arrows indicate the stimulated axillary buds. Scale bars = 1 cm. (B) Outgrowth of the buds at node 1 (n = 24). (C) Outgrowth of the buds at node 2 at 2 weeks after treatment (*n* > 20). Treatments were conducted at node 1 with GA_3_ (500 μM), BA (500 μM) or GA_3_ + BA. Values are means SE. Student’s *t*-test was used to determine significant differences between the treated and control groups. Significance levels: **P < 0.01.

**Fig. 3:**
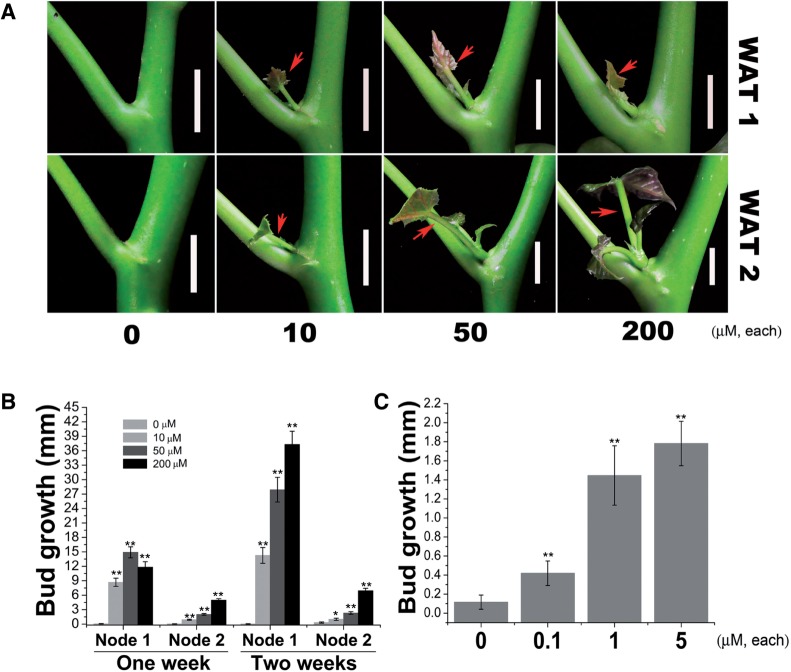
Co-application of GA_3_ and BA at low concentrations effectively promoted lateral bud outgrowth in 3-week-old seedlings of *J. curcas*. (A) Outgrowth of the buds at node 1 stimulated by GA_3_ + BA treatment. Photos were taken at 1 and 2 weeks after treatment (WAT 1 and 2). Red arrows indicate the stimulated axillary buds. Scale bars = 1 cm. (B) The length of the buds at nodes 1 and 2 was measured at 1 or 2 weeks after treatment with 10–200 μM GA_3_ + BA. (C) The length of the buds at nodes 1 was measured at 1 week after treatment with 0.1–5 μM GA_3_ + BA. Data are means ±SE (*n* = 20). Student’s *t*-test was used to determine significant differences between the treated and control groups. Significance levels: *P < 0.05; **P < 0.01.

As shown above, the lateral bud outgrowth in *J. curcas* seedlings can be stimulated by either GA_3_ or CK treatment; we thus evaluated the synergistic effect of these two hormones on the outgrowth of axillary buds in seedlings. Unlike on the adult *J. curcas* trees (Fig. 1A, B), bud treatment with only GA_3_ or BA had a very limited effect in promoting axillary buds in the seedlings to grow into complete branchlets, and the growth of the stimulated buds lasted only for 1 or 2 weeks (Fig. 2A). In contrast, co-application of the two phytohormones to buds revealed a significant synergistic effect on bud outgrowth in the seedlings (Fig. 2A, B). Axillary buds located at node 2 were also stimulated by the treatments (Fig. 2C), which may result from the hormone being transported up the stem after bud treatment at node 1. In addition, we found that the co-application of lower concentrations of GA_3_ and BA (e.g. 10 μM of each hormone) also successfully stimulated the outgrowth of axillary buds (Fig. 3). The promotive effect of the combined treatment of GA_3_ and BA at a low concentration (e.g. 10 μM of each, Fig. 3B) was greater than that of the separate treatment with GA_3_ or BA alone at a much higher concentration (500 μM of each, Fig. 2B). These results suggest that gibberellin and CK synergistically regulate lateral bud outgrowth in *J. curcas*.

**Fig. 4:**
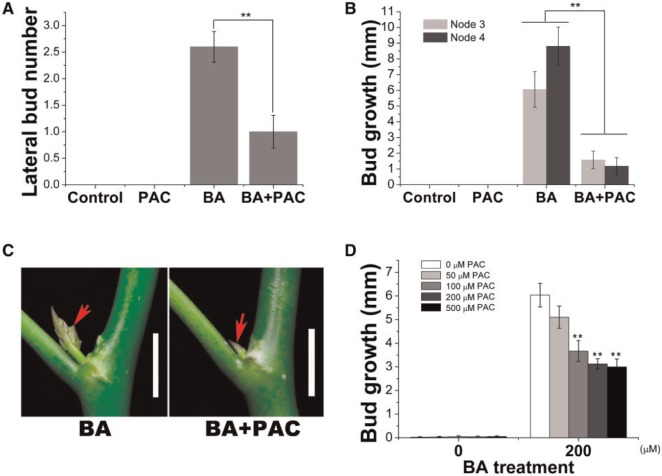
Paclobutrazol (PAC) inhibits promotion of bud outgrowth by BA. (A, B) PAC decreased the number of stimulated axillary buds and the outgrowth of buds in 4-week-old seedlings subjected to shoot treatment with BA (*n* > 15). The concentrations of PAC and BA used in single or combined treatments in (A) and (B) are 200 and 500 μM respectively. (C) Promotion of outgrowth of buds at node 1 by bud treatment with BA (200 μM) was inhibited by co-application of PAC (200 μM). Red arrows indicate the stimulated axillary buds. Scale bars = 1 cm. (D) Bud outgrowth of buds at node 1 by bud treatment with BA (200 μM) in conjunction with different concentrations of PAC (*n* = 20). All data and photos in (A–D) were taken at 1 week after treatment. Student’s *t*-test was used to determine significant differences between the treated and control groups. Significance levels: **P < 0.01.

We then investigated whether the inhibition of endogenous gibberellin biosynthesis could affect the promotion of bud outgrowth by BA in *J. curcas*. We found that the number of stimulated axillary buds was significantly reduced when paclobutrazol (PAC), an inhibitor of gibberellin biosynthesis (Ghosh et al. 2010, Ghosh et al. 2011), was co-applied with BA (Fig. 4A). The bud outgrowth promoted by BA was also severely inhibited upon PAC treatment (Fig. 4B, C), in a concentration-dependent manner (Fig. 4D). Since CK is the only phytohormone known to regulate shoot branching positively in the model plant pea (Domagalska and Leyser 2011, Janssen et al. 2014), we tested whether gibberellin also promoted shoot branching in pea, and whether the inhibition of gibberellin biosynthesis also affected the BA-mediated promotion of lateral bud outgrowth in pea. In contrast to the marked increase in lateral bud formation observed in *J. curcas* plants treated with GA_3_ (Fig. 1), GA_3_ had no obvious effect on bud outgrowth in pea (Supplementary Fig. S2A, B). The addition of PAC did not significantly affect the BA-mediated promotion of lateral bud outgrowth at node 2 or 3 (Supplementary Fig. S2), suggesting that gibberellin is not required during the early stages of bud outgrowth promoted by CK in pea.

**Fig. 5:**
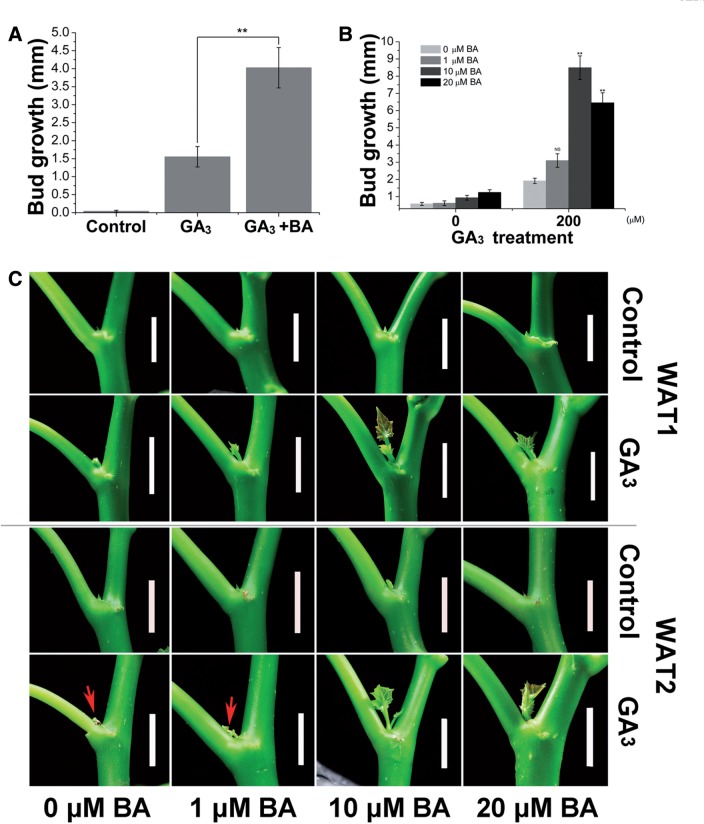
CK is required for promotion of bud outgrowth by gibberellin in the root-excised 3-week-old seedlings of *J. curcas*. (A) The length of buds at node 1 at 1 week after treatment with GA_3_ (200 μM) or GA_3_ (200 μM) + BA (20 μM) (*n* = 20). (B) Addition of various concentrations of BA in the liquid culture promoted the outgrowth of buds at node 1 at 1 week after GA_3_ treatment (*n* = 20). (C) Photographs of outgrowth of lateral buds at node 1 promoted by treatment with GA_3_ (200 μM) in conjunction with or without various concentrations of BA in the liquid culture. Red arrows indicated the buds that became dormant after 2 weeks growth. Scale bars = 1 cm. Root excision was conducted 2 d before hormone treatment. Values are means ± SE. Student’s *t*-test was used to determine significant differences between the treated and control groups. Significance levels: **P < 0.01; NS, not significant.

Since CKs, especially *trans*-zeatin (tZ), are produced mostly in the roots (Ko et al. 2014), we excised the roots of 3-week-old *J. curcas* seedlings to mimic immediate CK depletion, and cultured the shoots in the liquid medium to investigate further whether CK affects the bud promotion in *J. curcas* caused by GA_3_ treatment. The outgrowth of lateral buds in the root-excised seedlings of *J. curcas* was weakly stimulated by GA_3_ treatment, whereas co-application with BA or addition of BA in the aqueous culture significantly increased GA_3_-stimulated bud outgrowth (Fig. 5). Thus, a CK supply is required for the GA_3_-stimulated increase in bud formation in *J. curcas*.

### GA_3_, but not BA, induces lateral bud outgrowth on the newly developed shoots

**Fig. 6:**
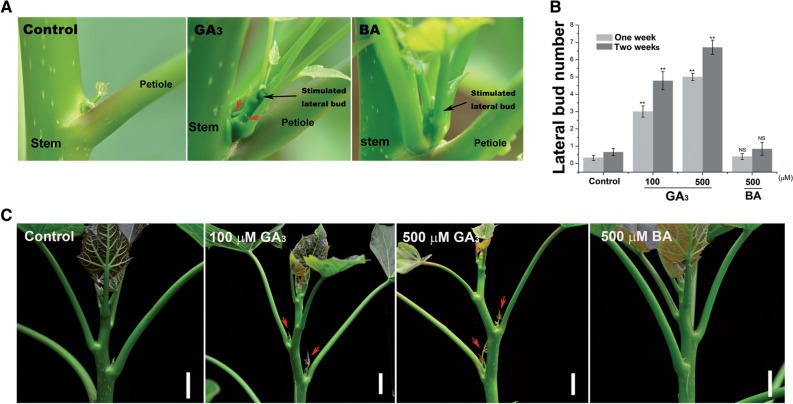
GA_3_, but not BA, induces lateral bud outgrowth on the newly developed shoots of 2-year-old *J. curcas* trees. (A) Secondary lateral buds (indicated by red arrows) were induced by treatment of shoots with GA_3_ (500 μM), but not BA (500 μM). (B) The number of lateral buds induced by treatment of shoot apex with GA_3_ or BA (*n* = 31–33). Values are means ± SE. Student’s *t*-test was used to determine significant differences between the treated and control groups. Significance levels: **P < 0.01; NS indicates no significant differences. (C) GA_3_ treatment at the shoot apex led to an obvious outgrowth of lateral buds (indicated by red arrows) on the newly developed shoots, while BA treatment did not. Scale bars = 1 cm.

As shown above, treatment of shoots with both GA_3_ and BA can effectively promote branching in *J. curcas*; however, GA_3_ treatment produced more branches than did BA treatment (Fig. 1A, B). We further found that GA_3_ induced formation of secondary buds in the axils of the newly developed lateral buds, whereas BA treatment did not (Fig. 6A). In addition, treatment with GA_3_, but not BA, at the shoot apex led to an obvious outgrowth of lateral buds on the newly developed shoots (Fig. 6C), which resulted in a significant increase in the number of lateral buds (Fig. 6B).

### GA_3_, BA and decapitation all influence the expression of putative branching regulators *JcBRC1* and *JcBRC2*

**Fig. 7:**
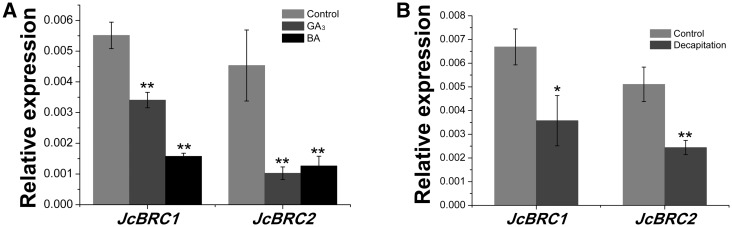
Effects of GA_3_, BA and decapitation on the expression of *JcBRC1* and *JcBRC2* at node 1 of 3-week-old *J. curcas* seedlings. (A) GA_3_ or BA (500 μM) treatment decreased the expression of *JcBRC1* and *JcBRC2* (*n* = 3). (B) Decapitation decreased the expression of *JcBRC1* and *JcBRC2* (*n* = 3). Decapitation was conducted on the 3-week-old seedlings by removing the shoot tip (approximately 1 cm). Samples for qPCR analysis were collected at 24 h after treatment. Values are means ± SE. Student’s *t*-test was used to determine significant differences between the treated and control groups. Significance levels: *P < 0.05; **P < 0.01.

Previous studies showed that *BRANCHED1* (*BRC1*) and *BRANCHED2* (*BRC2*) promoted bud arrest in response to environmental and endogenous signals, and that mutation of these genes caused ectopic shoot branching in Arabidopsis (Aguilar-Martínez et al. 2007). *BRC1* acts as a local integrator controlling bud outgrowth that is positively regulated by SLs, and down-regulated by CK treatment in Arabidopsis and pea (Aguilar-Martínez et al. 2007, Braun et al. 2012). We identified the homologs of *BRC1* and *BRC2* in *J. curcas*, *JcBRC1* and *JcBRC2* (GenBank accession Nos. KM454467 and KM454466), and analyzed their expression at 24 h after GA_3_, BA and decapitation treatment. We found that the expression of *JcBRC1* and *JcBRC2* declined within 24 h of treatment with GA_3_ or BA (Fig. 7A). Furthermore, *JcBRC1* and *JcBRC2* were also down-regulated within 24 h of decapitation (Fig. 7B). These results suggest that the promotion of lateral bud outgrowth by GA_3_, BA and decapitation treatment may be due to the inhibition of the expression of *JcBRC1* and *JcBRC2*. However, although the expression of *BRC1* and *BRC2* was significantly up-regulated by the branching suppressor GR24 (a synthetic SL) in pea (Braun et al. 2012), the expression of *JcBRC1* and *JcBRC2* was slightly but not significantly increased after 12 h of GR24 treatment (Supplementary Fig. S3), which is in agreement with findings in maize and rice (Minakuchi et al. 2010, Guan et al. 2012).

### Bud outgrowth promoted by gibberellin or CK is inhibited by SL

**Fig. 8:**
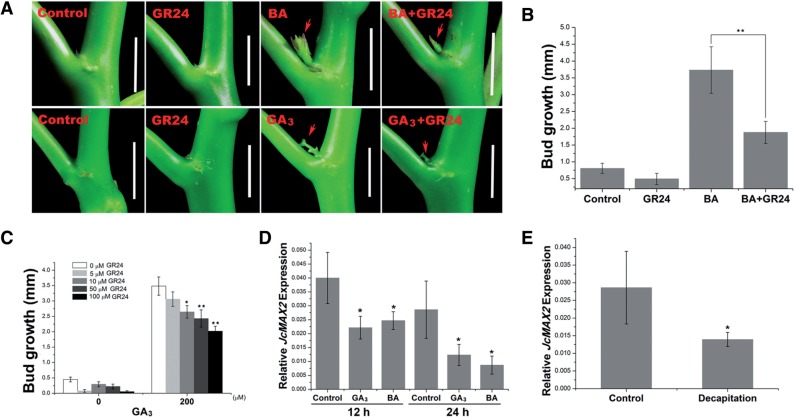
Strigolactones antagonistically regulates lateral bud outgrowth in *J. curcas* seedlings treated with GA_3_ and BA. (A–C) Bud outgrowth at node 1 of 3-week-old seedlings 1 week after GA_3_, BA, GR24, BA + GR24 or GA_3_ + GR24 treatments. Scale bars = 1 cm (*n* = 20). (D) GA_3_ and BA decreased the expression of *JcMAX2* at node 1 (*n* = 3). (E) Decapitation decreased the expression of *JcMAX2* at 24 h after treatment (*n* = 3). The concentration of BA, GA_3_ and GR24 used in (A), (B) and (D) was 200, 200 and 50 μM, respectively. Values are means ± SE for (B–E). Student’s *t*-test was used to determine significant differences between the indicated groups in (B) or between the treated and control groups in (C–E). Significance levels: *P < 0.05; **P < 0.01.

In pea, CK and SL antagonistically regulate bud outgrowth; exogenous application of the synthetic SL GR24 inhibits BA-stimulated bud formation in a dose-dependent manner (Dun et al. 2012). Here, we found that BA- or GA_3_-mediated bud promotion was significantly inhibited by GR24 in *J. curcas* (Fig. 8A–C). This result suggests that in *J. curcas* SL also acts antagonistically with gibberellin and CK in the control of lateral bud outgrowth. Arabidopsis *MORE AXILLARY GROWTH 2* (*MAX2*) plays a pivotal role in SL signaling transduction, and mutation of this gene results in an ectopic shoot branching phenotype (Stirnberg et al. 2002, Stirnberg et al. 2007). We showed that the expression of *JcMAX2*, a homolog of *MAX2* from *J. curcas* (GenBank accession No. KM454470), was decreased within 12 or 24 h of BA and GA_3_ treatment (Fig. 8D). In addition, *JcMAX2* expression was also down-regulated within 24 h of decapitation (Fig. 8E). These observations suggest that the down-regulation of *JcMAX2* expression could contribute to the promotion of bud outgrowth by gibberellin, CK and decapitation in *J. curcas*.

## Discussion

In this work, we report that gibberellin acts as a positive regulator of shoot branching in the woody plant *J. curcas*. We provided physiological and molecular evidence that gibberellin interacts with two other well-studied phytohormones, CK and SL, in the control of lateral bud outgrowth in *J. curcas.*

**Fig. 9:**
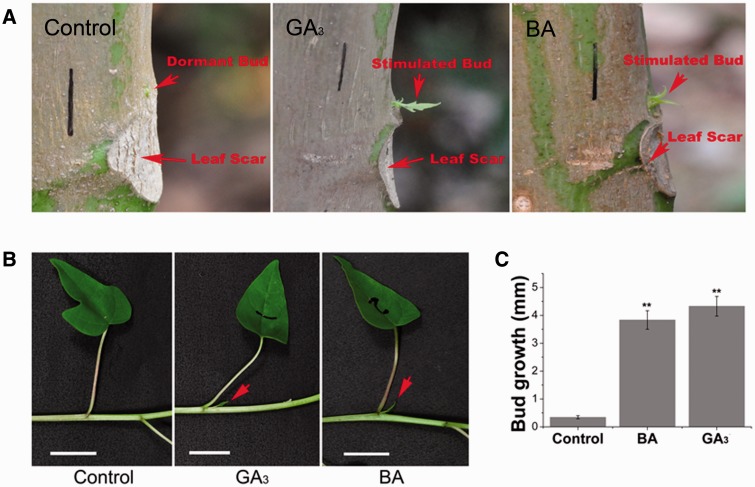
GA_3_ promotes lateral bud outgrowth in papaya. (A) One-year-old papaya tree. (B) Six-week-old papaya seedlings. (C) Bud length at node 2 of 6-week-old papaya seedlings was measured 3 weeks after GA_3_ or BA treatment (*n* = 32–34). The axillary buds of papaya were directly treated with GA_3_ (500 μM) or BA (500 μM) solution. Values are means ± SE for (C). Student’s *t-*test was used to determine significant differences between the treated and control groups. Significance levels: **P < 0.01.

**Fig. 10:**
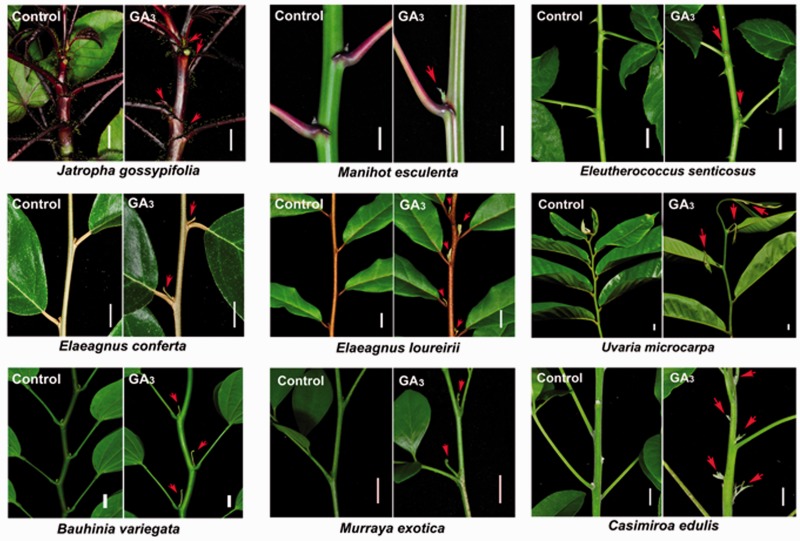
GA_3_ promotes the lateral bud outgrowth in other tree plants. The top approximately 20 cm of the selected branches was sprayed with 500 μM GA_3_ or the control solutions every other day twice. The red arrows indicate the stimulated lateral buds. Scale bars = 1 cm.

Although a negative correlation between gibberellin levels and branching or tillering has been observed in some species, such as Arabidopsis (Silverstone et al. 1997), pea (Scott et al. 1967, Luisi et al. 2011), rice (Lo et al. 2008, Qi et al. 2011), barley (Jia et al. 2009, Jia et al. 2011), turfgrass (Agharkar et al. 2007) and *Populus* trees (Mauriat et al. 2011, Zawaski and Busov 2014), we found that gibberellin acts as a positive regulator in the regulation of shoot branching in the perennial woody plant *J. curcas*, demonstrated by direct GA_3_ treatment (Fig. 1) and by overexpressing the gibberellin biosynthesis gene *JcGA20ox1* (Supplementary Fig. S1). On the basis of the results of this study, we hypothesize that gibberellin may function as a potent promoter of shoot branching in many perennial woody plants. Indeed, significant GA_3_-mediated promotion of bud outgrowth was also found in another woody plant, papaya (Fig. 9). By shoot treatment with GA_3_ and BA on the 2-year-old papaya trees, the outgrowth of the lateral buds on the stem was successfully stimulated (Fig. 9A). On the papaya seedlings, the lateral buds also showed strong response to GA_3_ or BA treatment (Fig. 9B, C). These results indicated that gibberellin was also involved in the positive regulation of bud outgrowth in papaya as it was in *J. curcas*. To investigate whether the positive role of gibberellin in the regulation of shoot branching is common in the perennial woody or shrub plants, we then conducted GA_3_ treatment on more tree species. It turned out that most of them showed accelerated outgrowth of lateral buds after GA_3_ treatment (Fig. 10). However, some species (e.g. *Bischofia javanica* and *Glochidion eriocarpum*) showed no obvious responses to GA_3_ (Supplementary Fig. S4), which is similar to the results we found in pea whereby GA_3_ treatment cannot promote bud outgrowth (Supplementary Fig. S2). It is possible that in those species, gibberellin may play a role as a negative factor in controlling lateral bud outgrowth as reported in pea (Scott et al. 1967, Luisi et al. 2011), Arabidopsis (Silverstone et al. 1997) and *Populus* (Mauriat et al. 2011, Zawaski and Busov 2014). Collectively, our results suggest that compared with the model plants Arabidopsis and pea, a more complex network regulates shoot branching in many perennial trees, in which gibberellin plays an important positive role.

SL, auxin and CK are three key phytohormones that co-ordinately regulate shoot branching. In pea and Arabidopsis, CK is the only phytohormone playing a positive role in regulating bud outgrowth, while gibberellin seems to inhibit this process. The lateral bud outgrowth is believed to be closely correlated with CK levels in the bud (Turnbull et al. 1997, Tanaka et al. 2006). After decapitation, the expression levels of CK biosynthesis genes (*IPT* genes) were rapidly increased at nodal stems, suggesting the locally biosynthesized CK rather than those derived from the roots controls bud outgrowth (Tanaka et al. 2006). Consistently in *J. curcas*, we also found that the expression level of *IPT* genes was rapidly increased at the nodal stems within 6 h after decapitation (Supplementary Fig. S5), which indicates that enhanced local CK biosynthesis is also a prerequisite for successful bud outgrowth in *J. curcas*. Initially, it was thought that bud outgrowth promoted by gibberellin in *J. curcas* could also be correlated with the increased CK biosynthesis at the nodal stem after GA_3_ treatment. However, the gene expression analysis at the nodal stems revealed that GA_3_ treatment significantly down-regulated the expression of most of the *IPT* genes (Supplementary Fig. S5), demonstrating that bud outgrowth induced by GA_3_ treatment is not through regulation of CK biosynthesis at the node, which is different from that by decapitation.

Our physiological results showed that both gibberellin and CK could effectively promote bud outgrowth in *J. curcas* and papaya. This raises the question of whether these two hormones act redundantly in the regulation of bud outgrowth in these species. In *J. curcas*, single GA_3_ or BA treatment can effectively promote bud outgrowth on the 2-year-old *J. curcas* trees (Fig. 1A), but the promotion is less effective on the 4-week-old seedlings (Fig. 2A). Unlike on the adult trees, growth of lateral buds on the seedlings after single GA_3_ or BA treatment only lasted for a few days, then the buds became dormant again (Fig. 2A), indicating that bud outgrowth was more tightly regulated on the seedlings than on the adult trees. The co-operation of gibberellin and CK in the regulation of bud outgrowth can be more obviously observed on the seedlings. Co-application of GA_3_ and BA had profound stimulative effects, which led to an enhanced and continued outgrowth of the lateral buds on seedlings (Fig. 2A, B). Co-application of GA_3_ and BA at much lower concentrations (e.g. 10 µM each) showed stronger stimulative effects on lateral bud outgrowth (Fig. 3B) compared with that by single GA_3_ or BA (500 µM) treatment (Fig. 2B). However, the successful outgrowth of the lateral buds severely inhibits the growth of apical buds (Supplementary Fig. S6), which probably resulted from competition for resources between lateral buds and apical buds. Moreover, we found that the bud outgrowth stimulated by BA treatment could be significantly inhibited by additional application of PAC (Fig. 4). However, we found that lateral buds of the root-excised shoots cultured in the liquid medium showed a weak response to GA_3_ treatment, whereas co-application with BA or addition of BA in the liquid medium rescued the response. These results demonstrate that in *J. curcas*, especially in the seedlings, gibberellin and CK are both required for the successful bud outgrowth. However, further work will be needed to elucidate the mechanism of how gibberellin and CK interact with each other in the promotion of bud outgrowth.

The findings in this study show that gibberellin also interacts with SL to regulate shoot branching in *J. curcas*. Nakamura et al. (2013) showed that rice *DWARF14* (*D14*), an essential component of plant SL signaling, interacts with a gibberellin signaling repressor *SLR1* in an SL-dependent manner, thus contributing to the negative regulation of gibberellin signaling, suggesting that SL and gibberellin signaling are co-ordinated during the control of shoot development and growth. It will be interesting to identify the mediators of the cross-talk between gibberellin and SL and other regulators of shoot branching in woody plants. Our findings indicate that the mechanism controlling shoot branching in perennial trees is not completely conserved with regard to that in annual species, emphasizing the importance of further studies to identify the components involved in the control of shoot branching in woody plants.

## Materials and Methods

### Plant materials and growth conditions

*Jatropha curcas* trees and seedlings were propagated from the cultivar ‘Flowery’. *Jatropha curcas* and *Carica papaya* (papaya) were grown in the experimental field and greenhouse at the Xishuangbanna Tropical Botanical Garden, Chinese Academy of Sciences (21°54'N, 101°46'E) (Pan et al. 2014). The photosynthetic active radiation reached 1,850 μmol m^−2^ s^−1^ in summer, and 1,550 μmol m^−2 ^s^−1^ in winter. The *J. curcas* seedlings used in the experiments were grown under long-day conditions (14 h light/10 h dark) at 22°C. The seedlings were grown in peat soil. Plants were fertilized with 1/4 Murashige and Skoog (MS) solution. For the root excision experiment, the shoots of 3-week-old seedlings were separated from the roots, leaving two-thirds of the hypocotyl tissues intact, and cultured in MS medium with or without BA in darkness overnight. Phytohormone treatments were administered at node 1 one day after the root excision. The excised stems were kept in an incubator (Sanyo) with 80% humidity, a 12 h light/12 h dark photoperiod, 25°C light/20°C dark and 80 μmol m^−2^ s^−1^ radiation.

### Phytohormone application

To make stock solutions (10 mM) of phytohormones, GA_3_ (Sigma) was dissolved in ethanol, BA (Sigma) in 0.5 M NaOH solution, and rac-GR24 (Chiralix) in acetone. These stock solutions were used to prepare working solutions of different concentrations, and the working solutions contained 0.05% (v/v) Tween-20 (BBI). All working solutions for each experiment had the same solvent. For the bud treatment, around 20 μl of working solution was directly dropped onto the leaf axil with a pipettor. For the shoot treatment, the stem was sprayed with working solution using a 100 ml plastic sprayer. Approximately 2 ml of working solution was used for each shoot treatment.

### *J. curcas* transformation

The full-length cDNA for *JcGA20ox1* was cloned into the binary vector pOCA30. Transformation of *J. curcas* was performed according to a previously described protocol (Pan et al. 2010). The transgenic shoots failed to generate roots in the root generation culture, so instead the transgenic shoots were grafted onto the rootstocks of wild-type *J. curcas* seedlings to generate transgenic plants (Li et al. 2014). The population of the transgenic lines was increased by grafting the transgenic shoots to the wild-type stock.

### RNA extraction and quantitative real-time PCR (qPCR)

For analysis of gene expression in the seedlings, the nodal stem at node 1 (approximately 10–20 mg) of 3-week-old *J. curcas* was sliced and immediately frozen in liquid nitrogen and kept at –80°C. For the expression profile analysis of gibberellin and SL biosynthesis genes, samples were collected from the 2-year-old *J. curcas* trees. All the samples were prepared in triplicate. Total RNA was isolated using pBIOZOL RNA extraction reagent (Bioer). RNA was quantified using a NanoDrop 2000 spectrophotometer (Thermo). Approximately 1 μg of total RNA was used for cDNA synthesis according to the method described in the TAKARA PrimeScript™ RT Reagent Kit (TAKARA Biotechnology). qPCR was performed on the LightCycler 480II (Roche). Primers are listed in Supplementary Table S1.

## Supplementary data

Supplementary data are available at PCP online.

## Funding

This work was supported by the National Natural Science Foundation of China [31370595]; the Top Science and Technology Talents Scheme of Yunnan Province [2009CI123]; the Natural Science Foundation of Yunnan Province [2011FA034]; the CAS 135 Program [XTBG-T02].

## Supplementary Material

Supplementary Data

## Abbreviations

BA: 6-benzyladenine
CK: cytokinin
IPT: adenosine phosphate isopentenyl transferase
MS: Murashige and Skoog
PAC: paclobutrazol
qPCR: quantitative real-time PCR
SL: strigolactone
tZ: *trans*-zeatin
