# Supplementary material for: Gibberellin Promotes Shoot Branching in the Perennial Woody Plant Jatropha curcas
Source: Plant Cell Physiol. 2015 Jun 15;56(8):1655–66. doi: 10.1093/pcp/pcv089 (PMC4523387; doi:10.1093/pcp/pcv089)
Supplement: Supplementary Data [file supp_56_8_1655__index.html]

Gibberellin promotes shoot branching in the perennial woody plant Jatropha curcas — Gibberellin Promotes Shoot Branching in the Perennial Woody Plant Jatropha curcas — Gibberellin Promotes Shoot Branching in the Perennial Woody Plant Jatropha curcas — Supplementary Data 

# Gibberellin Promotes Shoot Branching in the Perennial Woody Plant *Jatropha curcas*

## Supplementary Data

files

- Supplementary Data - pdf file
