# Supplementary material for: Gibberellin Promotes Shoot Branching in the Perennial Woody Plant Jatropha curcas
Source: Plant Cell Physiol. 2015 Jun 15;56(8):1655–66. doi: 10.1093/pcp/pcv089 (PMC4523387; doi:10.1093/pcp/pcv089)
Supplement: Supplementary Data [file supp_pcv089_pcp-2015-e-00126-File012.pdf]

Supplementary data

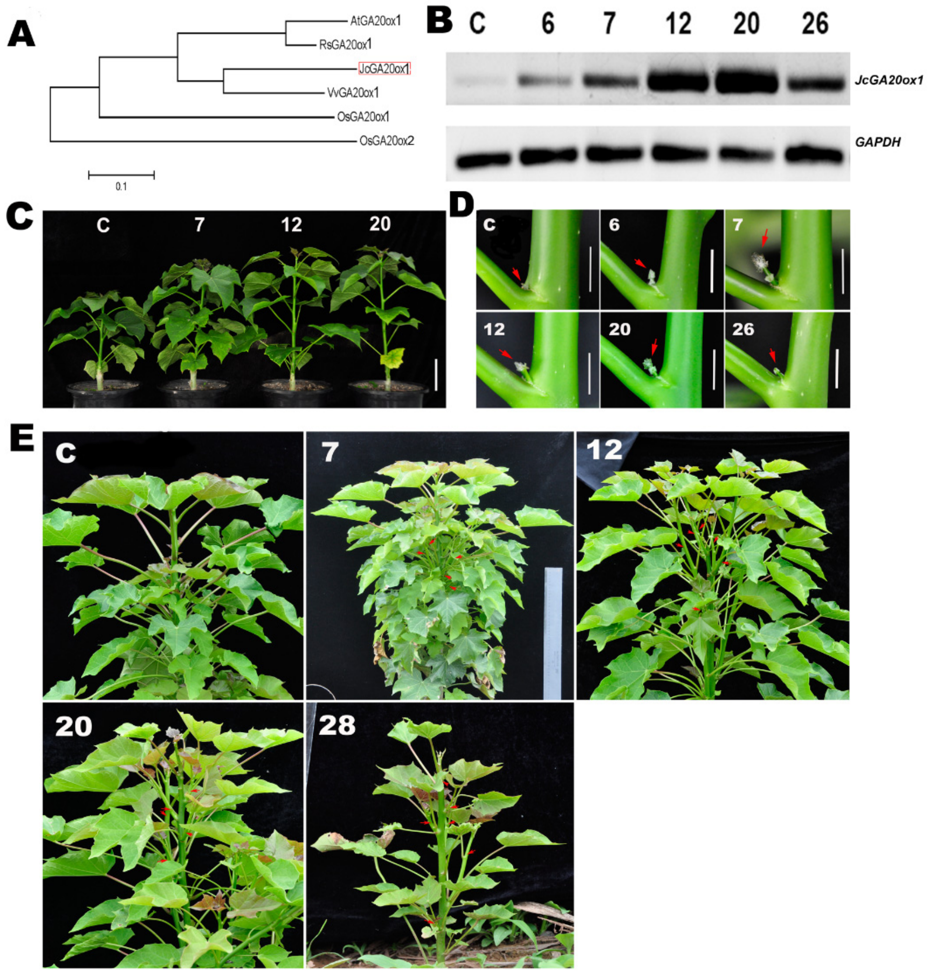

Supplementary Figure S1. Overexpression of *JcGA20ox1* in *J. curcas* promotes lateral bud outgrowth. (*A*) Phylogenetic analysis of JcGA20ox1 and its homologues from *Arabidopsis thaliana* (AtGA20ox1), *Ricinus communis* (RsGA20ox1), *Vitis vinifera* (VvGA20ox1), and *Oryza sativa* (OsGA20ox1 and OsGA20ox2). The scale represents 0.1 amino acid substitutions per site. (*B*) RT-PCR test for identification of positive transgenic lines. *JcGAPDH* is shown as control. (*C*) Control and transgenic plants overexpressing *JcGA20ox1* three months after grafting. Bars = 5 cm. (*D*) Overexpression of *JcGA20ox1* led to lateral bud outgrowth. Bars = 1 cm. (*E*) Transgenic plants in the field exhibited enhanced branching. (*B*) to (*E*): C indicates control plants, and the numbers indicate transgenic lines 6, 7, 12, 20, 26, and 28, respectively. The red arrows indicate the stimulated axillary buds.

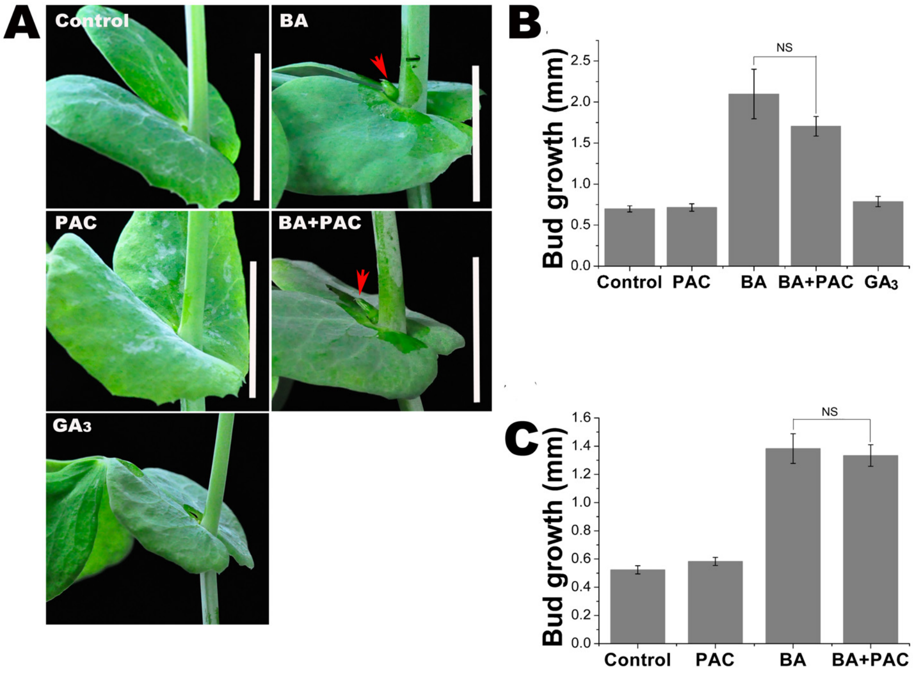

Supplementary Figure S2. Bud outgrowth in pea is significantly promoted by BA not GA treatment. (A and B) Outgrowth of lateral buds at node 3 was significantly affected by treatment with BA (100 μM), but not GA_3 (100 μM) or its biosynthesis inhibitor PAC (100 μM). Photos and data were taken at 5 days after treatment. (C) The BA-mediated increase in outgrowth of lateral buds at node 2 is not affected by co-application of PAC. Data were taken at 4 days after treatment. Two-week-old pea seedlings were used for all treatment in (A-C). In (B) and (C), values are means ± SE (n = 15). Student's *t*-test was used to determine significant differences between the indicated groups. NS indicates no significant difference.

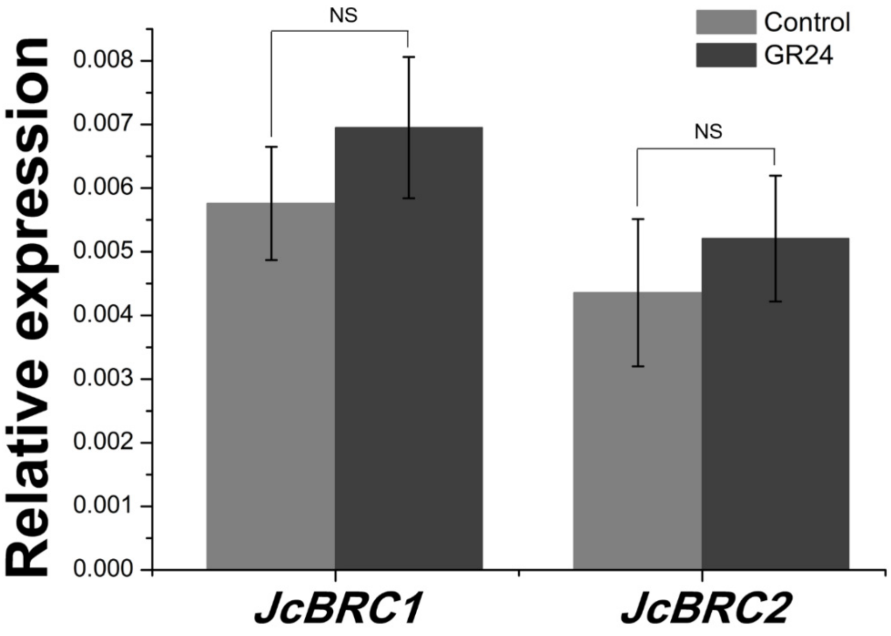

Supplementary Figure S3. Expression of *JcBRC1* and *JcBRC2* was not significantly affected by a 12-h GR24 (50 μM) treatment at node 1 of three-week-old *J. curcas* seedlings. *JcGAPDH* was used as the internal reference. Values are means ± SE (n = 3). Student's *t*-test (p value < 0.05) was used to determine significant differences between the treated and control groups. NS indicates no significant difference.

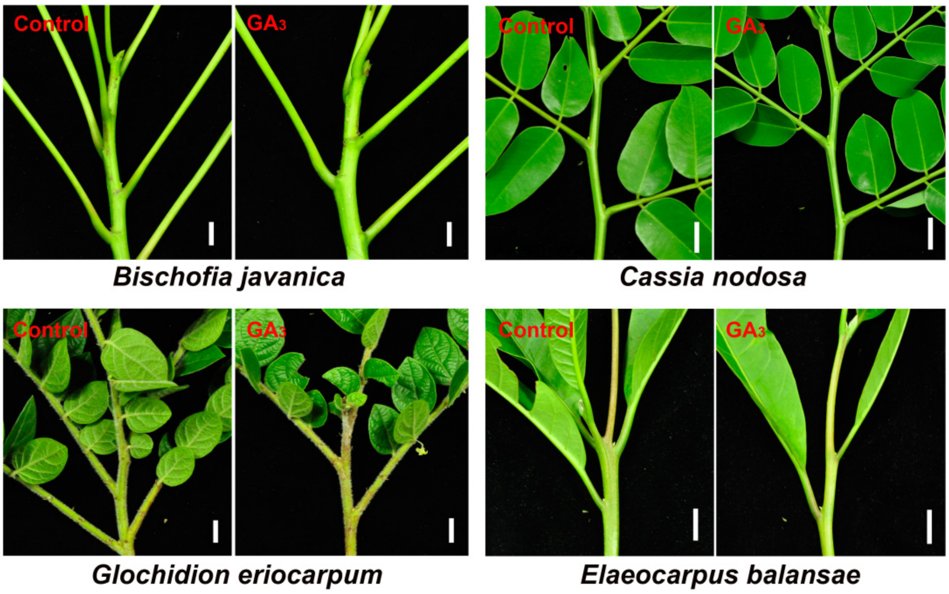

Supplementary Figure S4. GA_3 treatment did not promote the lateral bud outgrowth in *Bischofia javanica*, *Cassia nodosa*, *Glochidion eriocarpum* and *Elaeocarpus balansae*. Photos were taken one week after treatment. Bars = 1 cm.

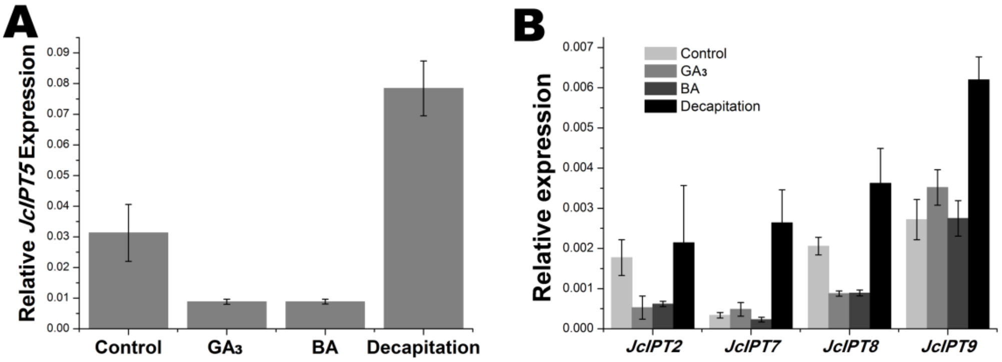

Supplementary Figure S5. Effects of GA_3, BA and decapitation on the expression of *JcIPT*s at node 1. The expression of *J. curcas* homologues of *Arabidopsis IPT5* (A), *IPT2*, *IPT7*, *IPT8*, and *IPT9* (B) was analyzed six hours after GA_3 (500 μM), BA (500 μM) or decapitation treatment. Values are means ± SE (n = 3). *JcGAPDH* was used as the internal reference.

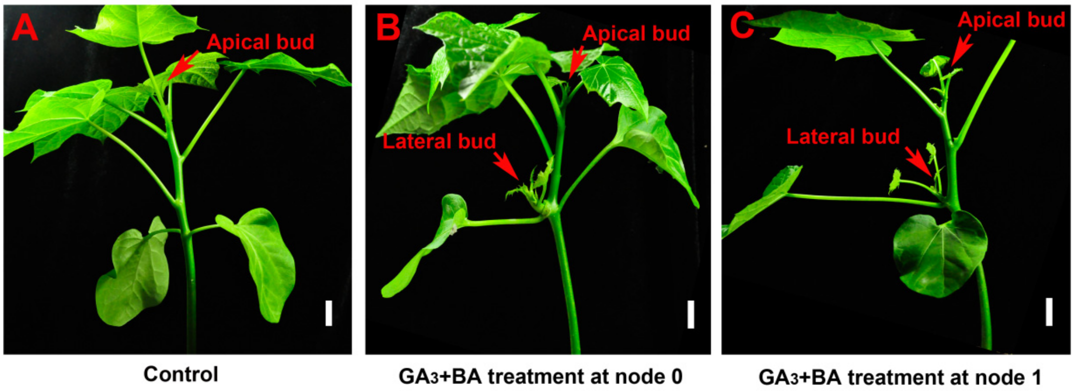

Supplementary Figure S6. Outgrowth of lateral buds inhibited the growth of apical buds. The outgrowth of lateral buds at node 0 (B) and node 1 (C) of three-week-old *J. curcas* seedlings was promoted by co-application with GA_3 and BA (200 μM each), which retarded the growth of apical buds. Photos were taken two weeks after treatment. Bars = 1 cm.

Supplementary Table S1. List of primers used in this study

Primer name	Primer sequence	Gene	Experiment
XK918	5’ GGAGAGAGCCAAGGAGAAAGAGA 3’	*JcBRC1*	qPCR
XK919	5’ GTAGCAGCAGTGTGATGGTTGT 3’		
XK958	5’ GAATGAGATTATCGCTCCGAATTGCTA 3’	*JcBRC2*	qPCR
XK959	5’ ACATTGCTTCACTCTTGGAACACTATC 3’		
XA275	5’ TGAACCCAAGCAAGTCCACCTCT 3’	*JcCCD7*	qPCR
XA276	5’ GCAGCAGCCACATCTGTGAAGT 3’		
XA277	5’ AAGTTGGAGGCAGCATTGGACC 3’	*JcCCD8*	qPCR
XA278	5’ AAGAGGTTCAGAAGGCACAGCAC 3’		
XK471	5’ CGGGATCCTTGATCTTCCTTTACCTAATGAATC 3’	*JcGA20ox-1*	CDS amplification
XK472	5’ AACTGCAGGGTTAACAGTGACATTATTATTAC 3’		
XA239	5' ACTTGGCGAACACTGTGGATATGC 3'	*JcGA20ox-1*	qPCR
XA240	5' ATTGCTCATCACCTCACTGTACTCCT 3'		
XT960	5' TGAAGGACTGGAGAGGTGGAAGAGC 3'	*JcGAPDH*	qPCR
XT961	5' ATCAACAGTTGGAACACGGAAAGCC 3'		
XA279	5' AGTGGTCGGTTAGATGAGATTGTAGC 3'	*JcMAX1*	qPCR
XA280	5' TGTGAACGCTGTTGTCGCTGAC 3'		
XA235	5’ GAGACGAGACGCTGCTTGCTATTG 3’	*JcMAX2*	qPCR
XA236	5’ CCTACAACACTGATCCGAGCATCCT 3’		
XT836	5' CCAATACCACCTCCTCgCCAC 3'	*JcIPT2*	qPCR
XT837	5' gACCCAACATCAAgACCACgg 3'		
XT834	5' ggCATCAAgCCTCggACTCTg 3'	*JcIPT5*	qPCR
XT835	5' AggCAATgACACATCCACCCA 3'		
XT846	5' TCAgTTCCACTCCCATTACATTT 3'	*JcIPT7*	qPCR
XT847	5' TgATTTACCACACCCAgTTgCTC 3'		
XT838	5' CAgAgTTCTTCAggAAgCAATACgC 3'	*JcIPT8*	qPCR
XT839	5' ggCTgTCAAACTgTAAAgAAACCg 3'		
XT977	5' ATTCAggCTCTggTgAgTCCATTCC 3'	*JcIPT9*	qPCR
XT978	5' TTCCACCATACTCAggCgTCTCA 3'		
